# Review of Melatonin's Effectiveness and the Side Effects on Alzheimer's Disease

**DOI:** 10.1192/bjo.2024.78

**Published:** 2024-08-01

**Authors:** Sui Yung Chen, Farooq Khan, Shoaib Talib, Suzanne Toft

**Affiliations:** 1 Black Country Healthcare NHS Foundation Trust, Sandwell, United Kingdom; 2 Birmingham and Solihull Mental Health NHS Foundation Trust, Birmingham, United Kingdom; 3 Black Country Healthcare NHS Foundation Trust, Walsall, United Kingdom

## Abstract

**Aims:**

People who have Alzheimer's disease (AD) often experience sleep disturbances due to the nature of the illness. Melatonin has been prescribed for sleep disturbance in individuals with AD, although there is a lack of national guidelines for pharmacological care for this presentation. Prolonged sleep disturbances for individuals with AD tend to lead to poor quality of life for the individual, behavioural challenges, carers' exhaustion and potential placement breakdowns.

The objective of this literature review is to determine whether the available evidence supports recommending melatonin to patients with AD for sleep, along with other benefits and adverse effects.

The hypothesis for this review is that melatonin is beneficial for sleep disturbances and has neuroprotection for individuals with AD.

**Methods:**

Literature search on the online electronic database from 2010 to November 2023, using the title of “Melatonin's effectiveness and the side effects on Alzheimer's Disease''. This literature review was done by screening the 125 searched titles. The inclusion criteria included systematic review (SR), meta-analysis, randomised controlled trial (RCT), animals and cell studies. Exclusion criteria included case studies, literature and peer reviews. A total of 12 papers are included in this review.

**Results:**

The three SRs, two meta-analyses and one RCT showed the potential effect of melatonin on ameriolating cognitive decline, improving cognition, quality of life and sleep qualities, with the conclusion that further studies are required. One combined meta-analysis and SR showed melatonin might be an effective treatment for mild AD. One Cochrane review showed melatonin has no evidence of improving sleep for moderate-to-severe AD.

One animal study and two cellular studies showed a melatonin effect in the control progression of AD. One animal study and one cellular research study concluded that melatonin has potential treatment effects.

Adverse effects were mentioned at the higher dose (10mg) with negative reaction times, sedation and confusion.

**Conclusion:**

There is a potential favourable effect of prescribing melatonin for mild to moderate AD, but there is limited evidence for prescribing it for moderate to severe AD. Furthermore, there is emerging evidence on melatonin's neuroprotective effect and potential treatment options for mild to moderate AD; further research is required for both sleep and neuroprotection in AD.

